# Neurogenesis Potential Evaluation and Transcriptome Analysis of Fetal Hypothalamic Neural Stem/Progenitor Cells With Prenatal High Estradiol Exposure

**DOI:** 10.3389/fgene.2021.677935

**Published:** 2021-06-22

**Authors:** Huihui Wang, Chengliang Zhou, Min Hou, Hefeng Huang, Yun Sun

**Affiliations:** ^1^Center for Reproductive Medicine, Renji Hospital, School of Medicine, Shanghai Jiao Tong University, Shanghai, China; ^2^Shanghai Key Laboratory for Assisted Reproduction and Reproductive Genetics, Shanghai, China; ^3^Animal Laboratory, Renji Hospital, School of Medicine, Shanghai Jiao Tong University, Shanghai, China; ^4^International Peace Maternity and Child Health Hospital, School of Medicine, Shanghai Jiao Tong University, Shanghai, China; ^5^Shanghai Key Laboratory of Embryo Original Diseases, Shanghai, China; ^6^Hospital of Obstetrics and Gynecology, Fudan University, Shanghai, China

**Keywords:** prenatal exposure, estradiol, neural stem/progenitor cells, neurogenesis, RNA sequencing, gene set enrichment analysis, protein-protein interaction, interaction network

## Abstract

High maternal estradiol is reported to induce metabolic disorders by modulating hypothalamic gene expression in offspring. Since neurogenesis plays a crucial role during hypothalamus development, we explored whether prenatal high estradiol exposure (HE) affects proliferation and differentiation of fetal hypothalamic neural stem/progenitor cells (NSC/NPCs) in mice and performed RNA sequencing to identify the critical genes involved. NSC/NPCs in HE mice presented attenuated cell proliferation but increased neuronal differentiation *in vitro* compared with control (NC) cells. Gene set enrichment analysis of mRNA profiles indicated that genes downregulated in HE NSC/NPCs were enriched in neurogenesis-related Gene Ontology (GO) terms, while genes upregulated in HE NSC/NPCs were enriched in response to estradiol. Protein-protein interaction analysis of genes with core enrichment in GO terms of neurogenesis and response to estradiol identified 10 Hub mRNAs, among which three were potentially correlated with six differentially expressed (DE) lncRNAs based on lncRNA profiling and co-expression analysis. These findings offer important insights into developmental modifications in hypothalamic NSC/NPCs and may provide new clues for further investigation on maternal environment programmed neural development disorders.

## Introduction

The theory that the intrauterine environment can influence prenatal development and the future health of offspring (Bateson et al., 2004) has resulted in increased interest in the developmental origin of chronic disease. Ovulation induction clinically used in assisted reproductive technology generates a supraphysiologic level of blood estradiol, which may predispose offspring to an abnormal intrauterine environment after fresh embryo transfer (Hu et al., 2014). Our previous study reported a programming effect by high maternal estradiol during early pregnancy on the hypothalamic glucoregulatory system of male mouse offspring, which induces adult metabolic disorders (Wang et al., 2018). This discovery indicates that high prenatal estradiol levels alter hypothalamus development, though the underlying mechanism is not yet well understood.

Neural stem/progenitor cells (NSC/NPCs) generate neurons during a process called neurogenesis (Bond et al., 2015). In the central nervous system (CNS), neurogenesis begins in the embryonic stage and continues throughout life. NSC/NPCs are preprogrammed to form specific types of functional neurons even before birth (Fuentealba et al., 2015); therefore, the study of prenatal neurogenesis may help researchers understand the mechanisms underlying adult neural disorders.

The hypothalamus regulates the metabolic homeostasis of the whole body and is sensitive to adverse prenatal environment (Ralevski and Horvath, 2015), so the development of hypothalamic neurons could affect metabolism in later life. Hypothalamic NSC/NPCs are first generated between embryonic day 10.5 (E10.5) and E14.5 in rodents (Padilla et al., 2010), and can proliferate to form neurospheres and differentiate into neurons *in vitro* (Desai et al., 2011). Substantial evidence has shown that dysfunctions of hypothalamic NSC/NPCs are associated with metabolic disorders, such as obesity and glucose intolerance (Li et al., 2012, 2014; Livesey, 2012), but the corresponding whole genomic features of these NSC/NPCs are rarely reported.

RNA sequencing (RNA-Seq) is an effective approach to revealing genome alterations which provides expression information for all transcripts, including mRNAs and non-coding RNAs. LncRNAs may play a considerable regulatory role by interacting with mRNAs, so exploring the link between them may provide more clues for elucidating molecular pathways. Gene set enrichment analysis (GSEA) is a robust and tractable analytical method for interpreting RNA-Seq data, as it can detect differential enrichment of biological functions across an entire network of genes (Subramanian et al., 2005), without the limitations associated with the single-gene method.

Because a prenatally programmed adult hypothalamic disorder resulting from high maternal estradiol has been identified (Wang et al., 2018), here we explore whether neurogenesis potential is affected in fetal hypothalamic NSC/NPCs and attempt to identify the key lncRNA-mRNA network through integrated bioinformatic analysis. These findings may help us to understand molecular modifications of fetal hypothalamic neurogenesis resulting from an adverse intrauterine environment.

## Materials and Methods

### Animal Model and 5-Bromodeoxyuridine Labeling in Fetal Brain

A mouse model of prenatal high estradiol exposure was created based on our previously published method (Wang et al., 2018). Briefly, 8-week-old pregnant C57BL/6 mice received 100 μg/kg/d estradiol valerate [Sigma; high estradiol (HE) group] or an equal amount of blank solvent [corn oil; control (NC) group] *via* gavage from E5.5 to E11.5. For bromodeoxyuridine (BrdU) labeling, pregnant mice at E14.5 received a single intraperitoneal injection of 100 mg/kg BrdU (Sigma) 2 h before euthanized; then, the fetuses were extracted and decapitated. Male fetuses were identified by visual identification of testes next to the bladder using a dissection microscope. The brains of male fetuses were removed and fixed in 4% paraformaldehyde (PFA) for 24 h and then infiltrated with 20–30% sucrose. Brain sections of 20 μm were made using a freezing microtome (Leica) for immunofluorescence staining.

### Tissue Immunofluorescence

Brain sections were blocked with 5% bovine serum albumin/0.3% Triton X-100 for 1 h at room temperature and incubated with primary antibodies mouse anti-Nestin (1:200, Millipore, catalog no. MAB353) and rabbit anti-BrdU (1:100, Abcam, catalog no. ab152095) overnight at 4°C, followed by reaction with secondary antibodies anti-rabbit Alexa Fluor 488 (1:200, Invitrogen, catalog no. A-11008) and anti-mouse Alexa Fluor 594 (1:200, Invitrogen, catalog no. A-11005) for 2 h at room temperature before counterstaining with 4',6-diamidino-2-phenylindole. The BrdU^+^Nestin^+^ cells were counted in five serial sections across the hypothalamus in each mouse.

### NSC/NPC Isolation and Neurosphere Assay

The brains of E14.5 male fetuses were dissected quickly on ice to remove the hypothalami, which were then fragmented in Neurobasal-A (Gibco), digested with TrypLE (Gibco) in 37°C for 15 min, and gently triturated into single cells with tips. The cells were then washed twice in Neurobasal-A and suspended in a proliferation medium containing Neurobasal-A, 2% B27 (Gibco), 10 ng/ml EGF (PeproTech), 10 ng/ml bFGF (PeproTech), and 1% GlutaMAX (Gibco), seeded in ultralow adhesion 6-well plates at a density of 10^5^/ml (Li et al., 2012), and incubated in 5% CO_2_ at 37°C. The neurospheres were photographed under a microscope for 4 days (Marshall et al., 2007), and the number was counted and diameter measured using the software ImageJ on the fourth day after isolation.

### NSC/NPC Proliferation and Differentiation Assay

To assess the proliferation ability of NSC/NPCs, the primary neurospheres were digested to count single cells and passaged at a density of 10^5^/ml in ultralow adhesion 6-well plates. The total cell number in each of the first four passages was calculated based on the assumption that all of the cells from the previous passage were replated. For the BrdU incorporation assay, primary NCS/NPCs were plated on Matrigel (BD)-coated coverslips at a density of 10^5^/ml in proliferation medium and cultured for 24 h. Then, the cells were treated with 10 μm BrdU for 2 h before immunofluorescence staining.

For induced differentiation, primary NSC/NPCs were seeded as single cells at a density of 3 × 10^5^/ml in Matrigel-coated coverslips placed in 24-well plates in differentiation medium containing Neurobasal-A, 2% B27, 1% fetal bovine serum (Gibco), and 1 μm retinoid acid (Sigma). The medium was changed every second day for 10 days, and then, the coverslips were removed to receive immunofluorescence detection of target neuron markers.

### NSC/NPC Immunofluorescence

For immunofluorescence staining of NSC/NPCs, neurospheres were moved using tips to seed on Matrigel-coated coverslips for 20 min before detection, and cells on coverslips were fixed with 4% PFA for 15 min and blocked with 5% bovine serum albumin/0.3% Triton X-100 for 1 h at room temperature. Cells were then incubated with primary antibodies rabbit anti-Sox2 (1:400, Cell Signaling Technology, catalog no. 23064), mouse anti-Nestin (1:200, Millipore, catalog no. MAB353), rabbit anti-BrdU (1:100, Abcam, catalog no. ab152095), or mouse anti-Tuj1 (1:200, Cell Signaling Technology, catalog no. 4466) overnight at 4°C and with secondary antibodies anti-rabbit Alexa Fluor 488 (1:200, Invitrogen, catalog no. A-11008), anti-mouse Alexa Fluor 594 (1:200, Invitrogen, catalog no. A-11005), or anti-mouse Alexa Fluor 488 (1:200, Invitrogen, catalog no. A-11001) for 2 h at room temperature before counterstaining with 4',6-diamidino-2-phenylindole. The BrdU/Nestin and Tuj1 positive cells were counted in each group.

### RNA-Seq Analysis

Three samples of first-passage NC and HE NSC/NPCs were harvested separately, each containing cells obtained from two mice. RNA was extracted with TRIzol (Invitrogen), its quality valued by spectrophotometer, and its integrity checked by Agilent 2,100 bioanalyzer. Total RNA was enriched by oligo beads, fragmented into small pieces, and reverse transcribed into cDNA. Second-strand cDNA was synthesized by DNA polymerase I with dUTP to construct a strand-specific library. The cDNA was then purified, end-repaired, poly A-added, and ligated to Illumina adaptor. The libraries were size-selected by agarose gel electrophoresis, PCR-amplified, and sequenced by Illumina NextSeq 500 by Personal Bio Co. (Shanghai, China).

The raw RNA-Seq data were filtered by removing low-quality and adaptor-related reads. The clean reads were then aligned to the mouse reference genome (10 mm) using Tophat2. Coding and non-coding transcripts were distinguished by Coding Potential Calculator, Coding-Non-Coding Index, and Pfam-scan. Non-coding RNAs with length >200 nt and exon number ≥2 were considered to be lncRNAs. Expression values were expressed as reads per kilobase per million reads. Differential expression analysis was conducted using DESeq2 (Love et al., 2014). LncRNAs and mRNAs with a log_2_ (fold change) ≥1 or ≤−1 and FDR < 0.05 were considered differentially expressed.

### Bioinformatics Analysis

We conducted enrichment analyses using GSEA with the standard procedure obtained from the GSEA Web site.^1^ The number of permutations was set to 1,000, and FDR < 0.25 with *p* < 0.05 was considered statistically significant. We downloaded gene sets needed for Kyoto Encyclopedia of Genes and Genomes (KEGG) and Gene Ontology (GO) analysis from the GSEA Web site. The enrichment bubble diagrams were made with R software (version 4.0.3).

A protein-protein interaction (PPI) network of selected mRNAs was constructed using STRING 11.0,^2^ and the Hub mRNAs [the top 10 nodes ranked by Maximal Clique Centrality (MCC); Chin et al., 2014] were identified using the plugin cytoHubba in Cytoscape software.^3^

To explore the lncRNA-mRNA regulatory network, Pearson’s correlation coefficient (PCC) between DE lncRNAs and Hub mRNAs was calculated and plotted using R software, and gene pairs with PCC ≥0.990 or ≤−0.990 and *p* < 0.05 were considered to be potentially correlated. The interaction network was visualized using Cytoscape software.

### Quantitative Real-Time Polymerase Chain Reaction

Total RNA of NSC/NPCs was extracted using TRIzol (Invitrogen) and reverse-transcribed into cDNA using Primer Script RT Reagent Kit (Takara) and amplified with QuantiNova SYBR Green PCR Kit (QIAGEN) according to the manufacturer’s instructions. The thermocycling conditions were 95°C for 2 min, followed by 40 cycles of 95°C for 5 s and 60°C for 10 s. The primers are listed in Table 1. GAPDH was used as an endogenous control, and the relative expression level was analyzed using the 2^−ΔΔCT^ method.

**Table 1:** Primer sequences for quantitative real-time polymerase chain reaction (qPCR).

Gene	Primer type	Primer sequence
*Tbr1*	Forward	GCAGCAGCTACCCACATTC
	Reverse	GTCCTTGGAGTCAGGAAAATTGT
*Six3*	Forward	TCAACAAACACGAGTCGATCC
	Reverse	TGGTACAGGTCGCGGAAGT
*Foxg1*	Forward	GAAGGCCTCCACAGAACG
	Reverse	CAAGGCATGTAGCAAAAGAGC
*Pou3f2*	Forward	GCAGCGTCTAACCACTACAGC
	Reverse	GCGGTGATCCACTGGTGAG
*Dlx2*	Forward	GGCTCCTACCAGTACCACG
	Reverse	GTAGCCCAGGTCGTAGCTG
*Fezf2*	Forward	GCAAAGGCTTTCACCAAAAA
	Reverse	GCATGTGGAAGGTCAGATTG
*Dlx1*	Forward	ATGCCAGAAAGTCTCAACAGC
	Reverse	AACAGTGCATGGAGTAGTGCC
*Nkx2-1*	Forward	ATGAAGCGCCAGGCTAAGG
	Reverse	GGTTTGCCGTCTTTGACTAGG
*Sox1*	Forward	TTTTCCGGGGTTTACTTCC
	Reverse	GCTCGAGGTCCGTCACTC
*Notch1*	Forward	TGCCACAATGAGATCGGCTC
	Reverse	GGGCACATAGGGCAGTTCA
*Egfr*	Forward	ATGAAAACACCTATGCCTTAGCC
	Reverse	TAAGTTCCGCATGGGCAGTTC
*Fgfr1*	Forward	ACTCTGCGCTGGTTGAAAAAT
	Reverse	GGTGGCATAGCGAACCTTGTA
*Fgfr2*	Forward	GCCTCTCGAACAGTATTCTCCT
	Reverse	ACAGGGTTCATAAGGCATGGG
*Fgfr3*	Forward	CCGGCTGACACTTGGTAAG
	Reverse	CTTGTCGATGCCAATAGCTTCT
*Fgfr4*	Forward	GCTCGGAGGTAGAGGTCTTGT
	Reverse	CCACGCTGACTGGTAGGAA

### Statistical Analysis

Data aside from RNA-Seq and bioinformatics analysis were analyzed using the Statistical Package for Sciences Software, version 21.0 (IBM), and are presented as the mean ± standard error of the mean. Unpaired Student’s *t*-tests were used for comparisons between two groups, and *p* < 0.05 was considered statistically significant.

## Results

### Prenatal High Estradiol Affects Neurogenesis Potential of Fetal Hypothalamic NSC/NPCs

**Figure 1:**
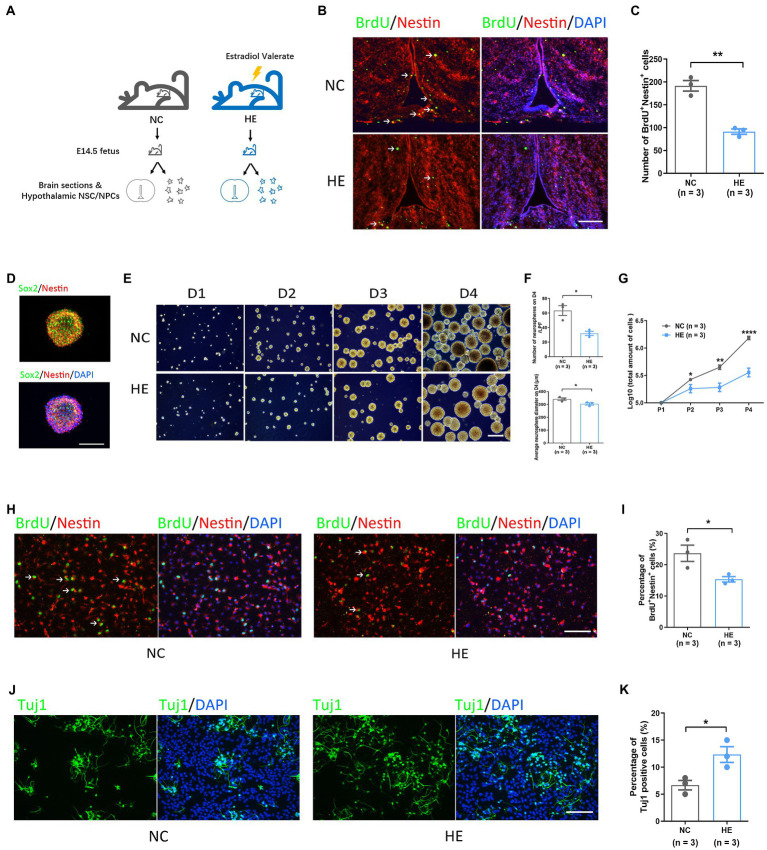
Isolation and evaluation of fetal hypothalamic neural stem/progenitor cell (NSC/NPC) neurogenesis potential in a mouse model. **(A)** Schematic of the method used to generate fetal brain sections and hypothalamic NSC/NPCs in a mouse model. **(B)** Representative images of bromodeoxyuridine (BrdU) and Nestin immunofluorescence in fetal hypothalamic tissue; scale bar: 200 μm. Arrows show BrdU^+^Nestin^+^ cells. **(C)** Quantification of BrdU^+^Nestin^+^ cells in five serial fetal hypothalamic tissue sections (*n* = 3 mice per group). **(D)** Representative images of Sox2 and Nestin immunofluorescence in neurospheres; scale bar: 100 μm. **(E)** Representative images of neurosphere formation across 4 days in the two experimental groups; scale bar: 400 μm. **(F)** Top: quantification of neurospheres on D4 of passage in two groups (*n* = 3 mice per group). Bottom: measurement of average diameters of neurospheres on D4 of passage in two groups (*n* = 3 mice per group). **(G)** Accumulated cell number of NSC/NPCs from P1 to P4 in the two experimental groups (*n* = 3 mice per group). **(H)** Representative images of BrdU and Nestin immunofluorescence in NSC/NPCs; scale bar: 100 μm. Arrows show BrdU^+^Nestin^+^ cells. **(I)** Quantification of BrdU^+^Nestin^+^ cells (%) in NSC/NPCs (*n* = 3 mice per group). **(J)** Representative images of Tuj1 immunofluorescence in neurons differentiated *in vitro* in the two experimental groups; scale bar: 100 μm. **(K)** Quantification of Tuj1 positive cells (%) in the two experimental groups (*n* = 3 mice per group). Error bars represent the standard error of the mean. Significance was determined by Student’s *t*-test. ^*^p < 0.05; ^**^p < 0.01; and ^****^p < 0.0001.

Serum estradiol after gavage in the HE pregnant mice reaches a peak value of four times that of the control group (Wang et al., 2018), forming a high maternal estradiol environment. Fetal brain sections were made on E14.5, and hypothalamic NSC/NPCs were isolated at the same time (Figure 1A). Authentic biomarkers Sox2 and Nestin were used to label NSC/NPCs (Suh et al., 2007; Gilyarov, 2008). Tissue immunofluorescence presented decreased number of BrdU^+^Nestin^+^ cells in HE fetal hypothalami after BrdU injection (Figures 1B,C), indicating a reduction of proliferating NSC/NPCs *in vivo*. Immunofluorescence staining of Sox2 and Nestin in neurospheres was performed for NSC/NPC identification after cell isolation (Figure 1D). The neurosphere assay showed a decreased amount of neurospheres with shorter average diameters in HE NSC/NPCs compared with NC on the fourth day (D4) of the first passage (Figures 1E,F), and the proliferation curve presented the accumulated NC NSC/NPC number significantly exceeded that in the HE group from the second to fourth passage (P2 to P4; Figure 1G), indicating attenuated proliferation ability in HE NSC/NPCs. We also performed BrdU incorporation assay in primary NSC/NPCs and found the decreased proportion of proliferating NSC/NPCs *in vitro* in HE group (Figures 1H,I).

NSC/NPCs from two groups were induced to differentiate into neurons, and the neuronic marker Tuj1 (Lee et al., 1990) was stained for quantification. The results showed neurons formed in both groups after a 10-day induction (Figure 1J); however, in contrast with proliferation assay, the number of neurons significantly increased in HE NSC/NPCs (Figure 1K).

### Transcriptional Analysis of NSC/NPCs Reveals Hub mRNAs Involved in Neurogenesis

**Figure 2:**
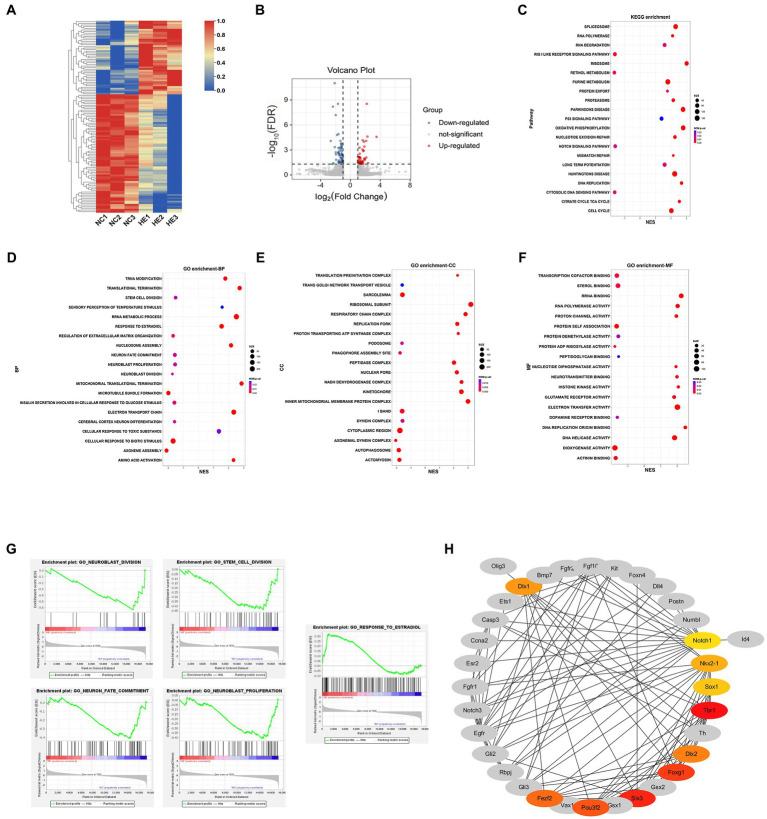
mRNA profiling and Hub mRNA identification. **(A)** Heatmap of 117 DE mRNAs in HE NSC/NPCs compared to NC. Red indicates upregulation, and blue indicates downregulation; row scale is from 0 to 1. **(B)** Volcano plot of DE mRNAs in HE NSC/NPCs compared with NC, red dots represent 45 upregulated mRNAs, and blue dots represent 72 downregulated mRNAs. **(C)** Kyoto Encyclopedia of Genes and Genomes (KEGG) enrichment results from gene set enrichment analysis (GSEA). The *x*-axis represents normalized enrichment score (NES), and the *y*-axis represents KEGG terms. The size of the dot indicates gene count, and the color indicates normalized value of *p*. Positive and negative NES indicate upregulation and downregulation in HE. **(D-F)** GO BP, CC (cellular component), and MF (molecular function) enrichment results from GSEA. The *x*-axis represents NES, and the *y*-axis represents GO terms. The size of the dot indicates gene count, and the color indicates normalized value of *p*. Positive and negative NESs indicate upregulation and downregulation in HE, respectively. **(G)** The enrichment plot of gene sets involved in neurogenesis and estradiol response. **(H)** Protein-protein interaction analysis of genes with core enrichment and Hub mRNA identification. Colored nodes indicate Hub mRNAs, with their shade positively correlated with the Maximal Clique Centrality score.

To elucidate the transcriptional changes related to altered neurogenesis, we compared the transcriptional profile of HE and NC NSC/NPCs by RNA-Seq. Heatmap of mRNAs showed distinctly different clustering between NSC/NPCs from two groups (Figure 2A). mRNAs with a log_2_ (fold change) ≥1 or ≤−1 and FDR < 0.05 were considered DE mRNAs, the volcano plot presented a total of 117 DE mRNAs, including 45 upregulated and 72 downregulated in HE NSC/NPCs compared with NC (Figure 2B). We conducted GSEA afterward, aiming to find neurogenesis-related gene sets (Figures 2C–F). The results revealed that genes downregulated in HE NSC/NPCs were enriched in neurogenesis-related GO biological processes (BP), such as neuroblast division, neuroblast proliferation, stem cell division, and neuron fate commitment, while genes upregulated in HE NSC/NPCs were enriched in response to estradiol (Figure 2D). The enrichment plots of these gene sets are presented in Figure 2G.

To further explore key mRNAs in the gene sets above and their interactions, mRNAs with core enrichment in each gene set (found in GSEA details) were picked for PPI analysis in STRING followed by Hub gene identification using Cytoscape. The top 10 genes ranked by MCC score were identified as Hub mRNAs, including *Tbr1*, *Six3*, *Foxg1*, *Pou3f2*, *Dlx2*, *Fezf2*, *Dlx1*, *Nkx2-1*, *Sox1*, and *Notch1* (Figure 2H).

**Figure 3:**
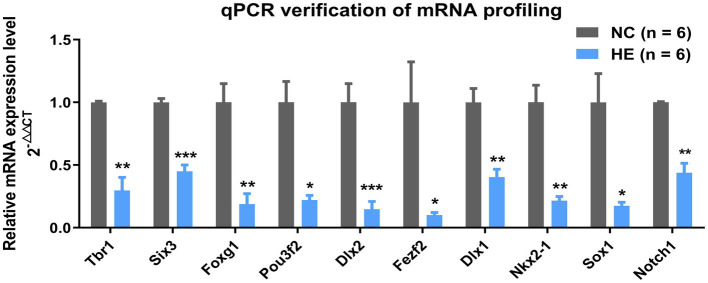
Verification of selected mRNAs by qPCR. Fold change of 10 Hub mRNAs in HE NSC/NPCs compared with NC (*n* = 6 mice per group). Significance was determined by Student’s *t*-test. ^*^p < 0.05; ^**^p < 0.01; and ^***^p < 0.001.

To validate the mRNA profiling and Hub gene identification results, the expression of the Hub mRNAs above was screened by quantitative real-time polymerase chain reaction with NSC/NPC samples used in RNA-Seq (six NC mice and six HE mice). The relative gene expression indicated all 10 Hub mRNAs decreased in the HE NSC/NPCs compared to the NC group and were identical to expression trends in RNA-Seq (Figure 3).

### Identification of DE lncRNA-Hub mRNA Interaction Network

**Figure 4:**
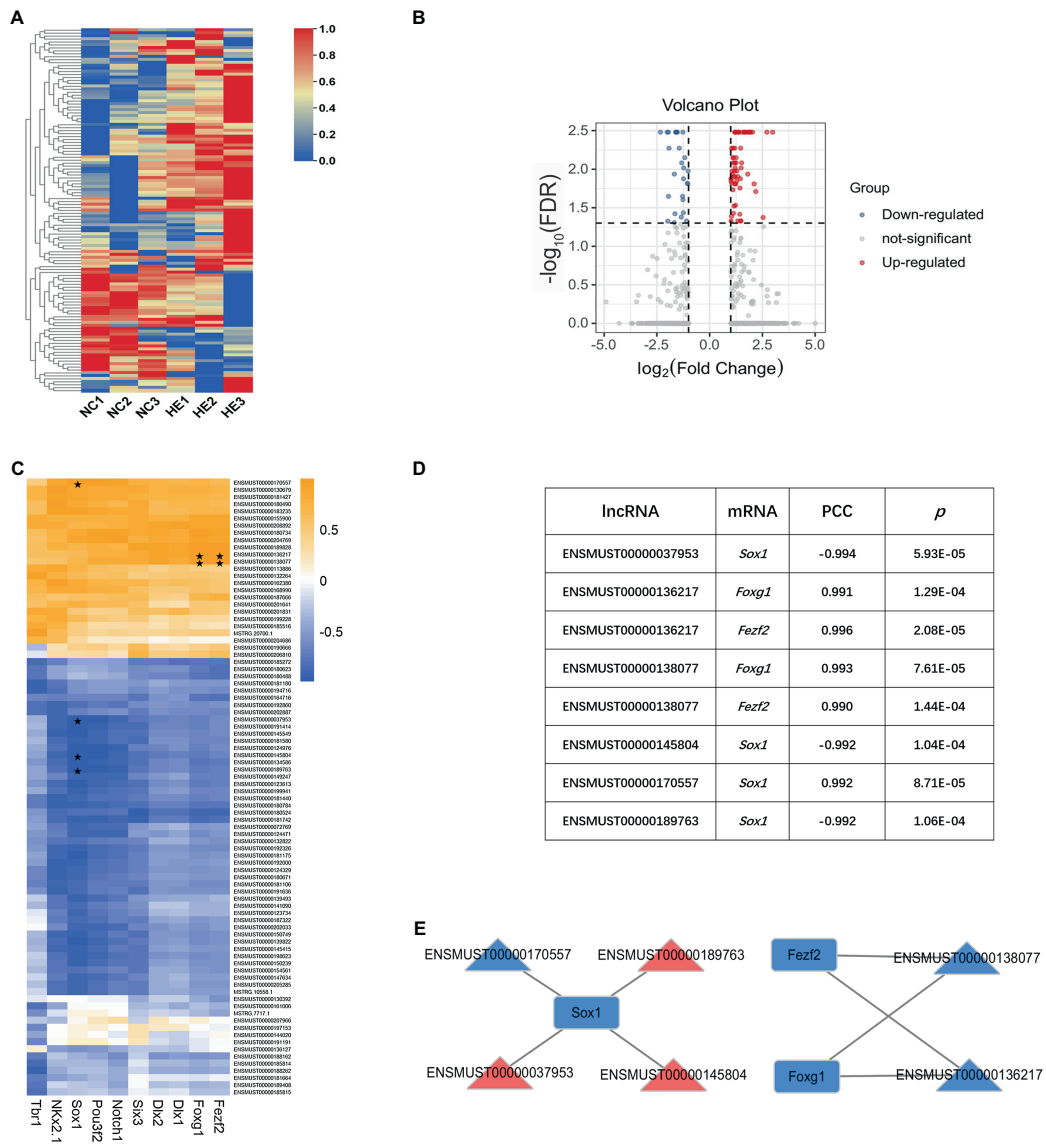
LncRNA profiling and DE lncRNA-Hub mRNA interaction network construction. **(A)** Heatmap of 85 DE lncRNA transcripts in HE NSC/NPCs compared to NC. Red indicates upregulation, and blue indicates downregulation; row scale is from 0 to 1. **(B)** Volcano plot of DE lncRNA transcripts in HE NSC/NPCs compared to NC. Red dots represent 58 upregulated transcripts, and blue dots represent 27 downregulated transcripts. **(C)** Heatmap of DE lncRNA-Hub mRNA Pearson’s correlation coefficient (PCC) score. Orange indicates positive PCC, and blue indicates negative PCC. PCC ≥0.990 or ≤−0.990 and *p* < 0.05 are labeled with ⋆. **(D)** List of correlated DE lncRNA transcript-Hub mRNA pairs, with their PCC and value of *p*. **(E)** The interaction network of six DE lncRNA transcripts and three Hub mRNAs. Red indicates upregulation, and blue indicates downregulation in HE NSC/NPCs compared to NC. Triangles represent lncRNAs, and rectangles represent mRNAs.

Epigenetic modification is recognized to regulate early life neurodevelopment (LaSalle et al., 2013; Yao et al., 2016), and dysregulation of lncRNAs leads to impaired development or neural dysfunction (Ng et al., 2013); therefore, we investigated whether lncRNA profiles were affected in NSC/NPCs with prenatal high estradiol stimulation. The resultant heatmap showed separated clustering of lncRNA transcripts in two groups (Figure 4A). Transcripts with a log_2_ (fold change) ≥1 or ≤−1 and FDR < 0.05 were considered DE lncRNA transcripts, and the volcano plot showed a total of 85 DE lncRNA transcripts, including 58 upregulated and 27 downregulated transcripts in the HE NSC/NPCs compared with the NC group (Figure 4B). The correlation between these DE lncRNA transcripts and the Hub mRNAs identified above were evaluated by constructing an expression matrix and calculating the PCC of each gene pair (Figure 4C). Gene pairs with a PCC ≥0.990 or ≤−0.990 and *p* < 0.05 were considered potentially correlated (Figure 4D). The co-expression network of these correlated genes was constructed (Figure 4E), including 6 lncRNA transcripts (ENSMUST00000037953, ENSMUST00000136217, ENSMUST00000138077, ENSMUST00000145804, ENSMUST 00000170557, and ENSMUST00000189763) and 3 Hub mRNAs (*Sox1*, *Fezf2*, *Foxg1*). Among them, ENSMUST00000189763, ENSMUST00000037953, and ENSMUST00000145804 were upregulated and the rest were downregulated. Therefore, we estimated these lncRNA-mRNA interactions may play a part in mediating the less proliferative and more neurogenic potential of fetal hypothalamic NSC/NPCs resulting from high maternal estradiol exposure.

## Discussion

Mounting evidence suggests that an adverse intrauterine hormonal environment could impair the health of offspring. High maternal estradiol is usually induced by ovarian stimulation in assisted reproductive technology, and it can disrupt neurodevelopment, resulting in metabolic disorders and diminished verbal abilities in offspring (Wang et al., 2018; Zhou et al., 2020). In our previous study, high maternal estradiol led to insulin resistance and disordered eating in mouse offspring due to decreased insulin receptor and elevated neuropeptide Y expression in the hypothalamus (Wang et al., 2018). That these alterations were detected postnatally prompted us to search for corresponding events in earlier developmental stages.

Because fetal NSC/NPCs proliferate and differentiate actively, they are vulnerable to exogenous stimulators like high maternal estradiol, which can pass through the placental barrier (Gude et al., 2004) and may directly affect their biological properties. We isolated primary neural stem cells from the fetal hypothalamus and cultured them as neurospheres. Actually, neurospheres may derive from mixed cells with dynamic markers, and even the purified cells remain heterogeneous (Pastrana et al., 2011), resulting in limitations in neurosphere assay if applied alone. Therefore, we examined proliferation in Sox2 and Nestin-positive cells both *in vivo* and *in vitro*, which are recognized to be NSCs and NPCs (Bani-Yaghoub et al., 2006; Ernst and Christie, 2006; Hernandez et al., 2007; Campbell et al., 2015). Since the vast majority of the primary cells were Sox2 and/or Nestin positive, as shown in Figures 1D,H, we consider the researches were performed mainly in the same population of cells in two groups. Although a small proportion of other type of cells might exist, their effect on gene expression appeared insignificant when compared with NCS/NPCs. In consideration of the limitations of our study, we believe single-cell RNA-Seq should make a more precise method in future research. In fact, the cell composition in neurospheres would possibly change during different passages, and we speculate that the diminished proliferation might be a continuation of properties in primary NSC/NPCs, and further studies are expected to reveal whether increased cell apoptosis and senescence could occur.

The proliferation medium contained EGF and bFGF, which are essential factors for NSC/NPC growth. To explore whether cell proliferation changed due to different levels of EGF and bFGF receptors, we examined the mRNA expressions of them in primary NSC/NPCs and found no significant difference between the two groups (Supplementary Figure S1). This result indicated that the decreased proliferation in HE NSC/NPCs was not attributed to different levels of growth factor receptors in the culture medium, but more likely to the intrauterine programming effect.

Neurogenesis is a complex process, and how NSC/NPCs are maintained, divided, and differentiated remain controversial (Lazutkin et al., 2019). Our results indicated decreased proliferative activity and increased neuronal production in HE NSC/NPCs; however, whether or not this resulted from premature exhaustion of the stem cell pool requires further study. A comprehensive evaluation of hypothalamic neurogenesis from the prenatal period to adulthood may show us a more precise effect of high maternal estradiol on neurodevelopment. It should be noted that the NSC/NPC differentiation assay was carried out *in vitro*, which generates early and immature neurons rather than functional neurons, and an *in vivo* labeling of NSC/NPCs at the embryonic stage followed by detections like lineage tracing may provide a more accurate indication of their differentiation directions.

Several published studies show that estrogen stimulates both proliferation and differentiation of NSC/NPCs (Okada et al., 2010; Li et al., 2020) and attenuates damage to neurogenesis in the developing brain caused by chemical drug exposure (Li et al., 2019); however, another study shows that 10 nM estradiol increases NSC/NPCs proliferation and stimulates differentiation into neurons *in vitro*, but 50 nM estradiol markedly decreases NSC/NPCs proliferation (Zhang et al., 2019a). Thus, the effects of maternal estradiol on neurogenesis of fetal hypothalamic NSC/NPCs may be dose dependent, and the dose that caused metabolic disorder in our mouse model exerted a different effect on proliferation and differentiation. The stimulative effect on neuron formation may explain our previous finding that hypothalamic neuropeptide Y increases in HE offspring (Wang et al., 2018); that is, prenatal high estradiol probably promotes orexigenic neuron generation, leading to disordered eating.

We used log_2_ (fold change) ≥1 or ≤−1 and FDR < 0.05 as the cutoffs to define DE genes in our study, and looser criteria of log_2_ (fold change) ≥1 or ≤−1 with *p* < 0.05 were also tried to identify DE genes and predict DE lncRNA-Hub mRNA regulatory network. This method presented 567 DE mRNAs (383 upregulated and 184 downregulated) and 148 DE lncRNA transcripts (89 upregulated and 59 downregulated) in HE NSC/NPCs compared with NC group (Supplementary Figures S2A–D). In spite of this, the potentially correlated DE lncRNA-Hub mRNA pairs remained unchanged compared with those marked in Figure 4C (Supplementary Figure S2E).

Gene set enrichment analysis (GSEA) revealed both enrichment and expression of mRNA profiles in two groups; positive NES indicated upregulation in HE compared with NC NSC/NPCs, and negative NES indicated downregulation. Although KEGG enrichment did not reveal items directly involved in cell proliferation and differentiation, it showed differential enrichment of cell cycles, DNA replication, nucleotide excision repair, and RNA polymerase. Moreover, we found upregulated mRNAs that are enriched in Huntington’s and Parkinson’s disease in the HE NSC/NPCs, suggesting an increased risk of neurodegenerative disease in offspring exposed to high maternal estradiol, which requires further validation. The GO CC and MF enrichment revealed downregulated genes enriched in autophagosome and dopamine receptor binding, and upregulated genes enriched in neurotransmitter binding and glutamate receptor binding in HE NSC/NPCs. These discoveries may help illuminate the mechanisms of maternal estradiol-induced neurodevelopmental disorders.

The GO BP enrichment result revealed downregulated genes in HE NSC/NPCs enriched in stem cell division, neuroblast proliferation, and neuroblast division, which consisted with their less proliferative potential observed. However, genes enriched in neuron fate commitment also decreased in HE NSC/NPCs, which seemingly contradicted the more neurogenic activity *in vitro*. To find a rational explanation for this, we focused on Hub mRNAs of core enrichment in these gene sets.

The Hub mRNAs were identified based on a PPI network consisting of genes enriched in neurogenesis and response to estradiol; however, the top 10 Hub mRNAs ranked by MCC in Cytoscape were all genes involved in neurogenesis, and they were all downregulated in HE NSC/NPCs. This result may be explained by the fact that Hub genes are highly connected genes in a co-expression network, and genes enriched in response to estradiol failed to present such close connections with those enriched in altered neurogenesis, implying a probable indirect effect of estradiol stimulation on neurogenesis in our study. Most of these Hub mRNAs are transcription factors except *Notch1*. *Pou3f2* influences multiple stages of neurogenesis by promoting neural transcription factor *Tbr1* (Dominguez et al., 2013), which regulates cell differentiation and migration and involves glutamatergic neurogenesis (Mihalas and Hevner, 2017). *Six3*, *Foxg1*, and *Sox1* maintain the balance between proliferation and neuronal differentiation of NSC/NPCs. Specifically, upregulation of *Six3* plays a role in keeping NSC/NPCs in an undifferentiated state (Appolloni et al., 2008); *Foxg1* deficiency leads to premature differentiation of neurons, and its overexpression increases the NSC/NPC pool (Hanashima et al., 2004; Brancaccio et al., 2010); and *Sox1* loss induces depletion of proliferating NSC/NPCs with increased cell cycle exit (Bylund et al., 2003). *Dlx1* and *Dlx2* drive GABAergic neuron generation (Lindtner et al., 2019; Barretto et al., 2020), and *Fezf2* is involved in the dopaminergic neuron generation (Eckler and Chen, 2014); moreover, knockdown of *Fezf2* leads to decreased *Foxg1* and *Six3* in mouse embryonic stem cells (Wang et al., 2011). *Notch1* signaling is reported to promote NSC/NPC proliferation but decrease neuronal differentiation during meningitis and spinal cord injury (Peng et al., 2019; Zhang et al., 2019b). *Nkx2-1* is a critical factor maintaining the anorectic gene *Pomc* expression from early development to adulthood (Orquera et al., 2019). To sum up, the published information above supports our findings that decreased *Six3, Foxg1*, *Sox1*, *Fezf2*, and *Notch1* in HE NSC/NPCs directly correlated with decreased proliferation and enhanced neuronal generation, which probably reflected a premature differentiation. As the upstream regulator of *Pomc*, decreased *Nkx2-1* in HE offspring could contribute to the verified orexigenic phenotype in later life (Wang et al., 2018). Since *Pou3f2*, *Tbr1*, *Dlx1*, and *Dlx2* are involved in the generation of glutamatergic or dopaminergic or GABAergic neurons, figuring out the neuron types that these NSC/NPCs tended to form would help validate the effect on neurogenesis of these genes in our experiment.

The published studies fail to specify the effect of estradiol on Hub mRNAs above during neurogenesis. One research shows estradiol stimulation does not affect *Notch1* expression during hippocampus development but reduces the level of its transcriptionally active domain (Bender et al., 2010). Since we previously found DNA methylation programs hypothalamic gene expression in HE offspring (Wang et al., 2018), it may support the hypothesis that expression changes of these Hub mRNAs could be attributed to epigenetic regulators, such as DNA methylation and non-coding RNAs.

LncRNAs are important components of regulatory networks in CNS development whose dysregulation leads to neurological disorders (Ng et al., 2013). Since lncRNAs exert functions mainly through regulating mRNA expression, we constructed the interaction network between DE lncRNAs and Hub mRNAs, aiming to discover lncRNAs possibly connected with the altered neurogenesis. Our study predicted six DE lncRNA transcripts correlated with three Hub mRNAs based on their expression level. These lncRNA transcripts (ENSMUST 00000037953, ENSMUST00000136217, ENSMUST 00000138077, ENSMUST00000145804, ENSMUST00000170557, and ENSMUST00000189763) are coded by genes *2810032 G03Rik*, *Prdm16os*, *Gm13110*, *Ppp1r18os*, *Gm17035*, and *D130058E05Rik*, respectively, according to Ensemble genome browser.^4^ We also checked the expression correlations between DE lncRNAs and all mRNAs in Figure 2H (mRNAs with core enrichment in neurogenesis and response to estradiol, core mRNAs for short), which predicted a potential regulatory network of 14 DE lncRNA transcripts and 11 core mRNAs (Supplementary Figures S3A–C). In consideration of our limited sample size, we repeated the co-expression analysis of these lncRNAs and mRNAs using the pubic dataset GSE65487 in Gene Expression Omnibus, which assessed the RNA profiles of proliferating progenitors, differentiating progenitors and neurons from E14.5 mouse cortex. However, five of DE lncRNAs were not detected in GSE65487 (Supplementary Figure S3D), and four of the rest presented potential correlations with three mRNAs each, when the cutoffs were set to be PCC ≥0.900 or ≤−0.900 and *p* < 0.05 (PCC ≥0.990 or ≤−0.990 identified no significant correlation; Supplementary Figures S3E,F). Although we failed to discover the same lncRNA-mRNA pair as demonstrated in our study by using this public dataset, it still reflected possible connections of these lncRNAs with neurogenesis. Since there are not yet any published literatures about the functions of these lncRNAs, future work focused on their specific roles is expected to help answer questions regarding development-originated neuroendocrine disorders.

In short, our research presents the cytologic changes in early neural development under a high maternal estradiol environment and reveals the corresponding whole genomic features with a prediction of the underlying molecular modifications. This study demonstrates comprehensive information about fetal hypothalamic NSC/NPCs with prenatal high estradiol exposure and contributes to our understanding of the fetal-programmed adult diseases.

## Data Availability Statement

The datasets presented in this study can be found in an online repository. The name of the repository and accession number can be found at https://www.ncbi.nlm.nih.gov/geo/query/acc.cgi?acc=GSE168075.

## Ethics Statement

This animal study was reviewed and approved by the Institutional Animal Care and Use Committee of Shanghai Jiao Tong University.

## Author Contributions

HW and CZ designed the experiments, collected the data, analyzed the data, and drafted the manuscript. MH, HH, and YS revised the final manuscript. All authors have reviewed the manuscript before submitting it and approved the final version.

### Conflict of Interest

The authors declare that the research was conducted in the absence of any commercial or financial relationships that could be construed as a potential conflict of interest.

## Footnotes

^1^http://www.gsea-msigdb.org/gsea/index.jsp

^2^https://string-db.org

^3^http://www.cytoscape.org

^4^http://asia.ensembl.org/index.html

## Supplementary Material

Supplementary Figure S1 mRNA expression of EGF and bFGF receptors examined by qPCR. Fold change of EGF and bFGF receptor mRNAs in HE NSC/NPCs compared with NC (*n* = 6 mice per group). Significance was determined by Student’s *t*-test; ns, not significant.

Supplementary Figure S2 DE gene identification and DE lncRNA-Hub mRNA interaction network analysis using log_2_ (fold change) ≥1 or ≤−1 with *p* < 0.05 as the cutoff. **(A)** Heatmap of 567 DE mRNAs in HE NSC/NPCs compared to NC. Red indicates upregulation, and blue indicates downregulation; row scale is from 0 to 1. **(B)** Volcano plot of DE mRNAs in HE NSC/NPCs compared to NC, red dots represent 383 upregulated mRNAs, and blue dots represent 184 downregulated mRNAs. **(C)** Heatmap of 148 DE lncRNA transcripts in HE NSC/NPCs compared to NC. Red indicates upregulation, and blue indicates downregulation; row scale is from 0 to 1. **(D)** Volcano plot of DE lncRNA transcripts in HE NSC/NPCs compared with NC. Red dots represent 89 upregulated transcripts, and blue dots represent 59 downregulated transcripts. **(E)** Heatmap of DE lncRNA-Hub mRNA PCC score. Orange indicates positive PCC, and blue indicates negative PCC. PCC ≥0.990 or ≤−0.990 and *p* < 0.05 are labeled with ⋆.

Supplementary Figure S3 DE lncRNA-core mRNA interaction network construction and verification with public dataset. **(A)** Heatmap of DE lncRNA-core mRNA PCC score. Orange indicates positive PCC, and blue indicates negative PCC. PCC ≥0.990 or ≤−0.990 and *p* < 0.05 are labeled with ⋆ (DE lncRNA-Hub mRNA) or ● (DE lncRNA-non Hub mRNA). **(B)** List of correlated DE lncRNA transcript-core mRNA pairs, with their PCC and value of *p*. **(C)** The interaction network of 14 DE lncRNA transcripts and 11 core mRNAs. Red indicates upregulation, and blue indicates downregulation in HE NSC/NPCs compared to NC. Triangles represent lncRNAs, and rectangles represent mRNAs. **(D)** List of lncRNAs in panel B with the corresponding lncRNA genes found in GSE65487. **(E)** Heatmap of lncRNA-mRNA PCC score graphed using data from GSE65487. Orange indicates positive PCC, and blue indicates negative PCC. PCC ≥0.900 or ≤−0.900 and *p* < 0.05 are labeled with ⋆. **(F)** List of correlated lncRNA-mRNA pairs presented in panel E, with their PCC and value of *p*.
