# Supplementary figures and images for: Neurogenesis Potential Evaluation and Transcriptome Analysis of Fetal Hypothalamic Neural Stem/Progenitor Cells With Prenatal High Estradiol Exposure
Source: Front Genet. 2021 Jun 22;12:677935. doi: 10.3389/fgene.2021.677935 (PMC8258253; doi:10.3389/fgene.2021.677935)

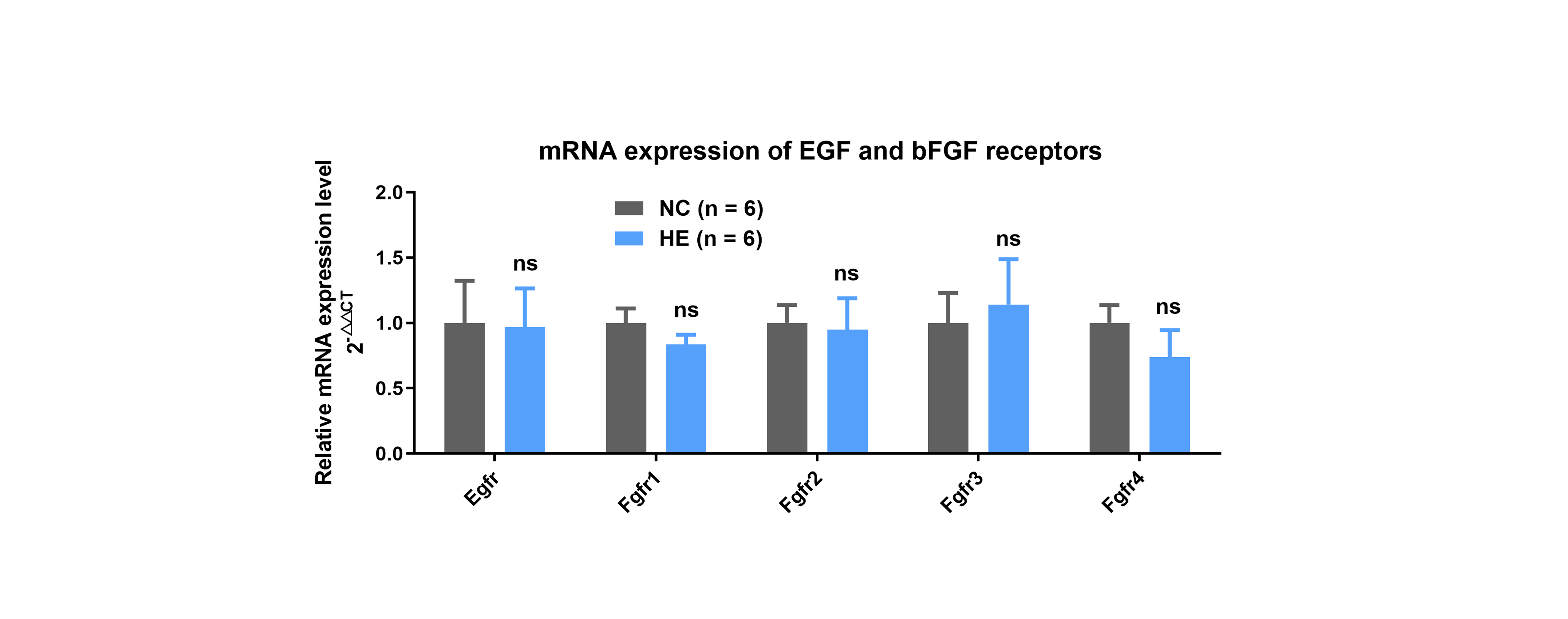

Supplement: Supplementary Figure S1 — mRNA expression of EGF and bFGF receptors examined by qPCR. Fold change of EGF and bFGF receptor mRNAs in HE NSC/NPCs compared with NC (n = 6 mice per group). Significance was determined by Student’s t-test; ns, not significant. [file Image_1.tif]

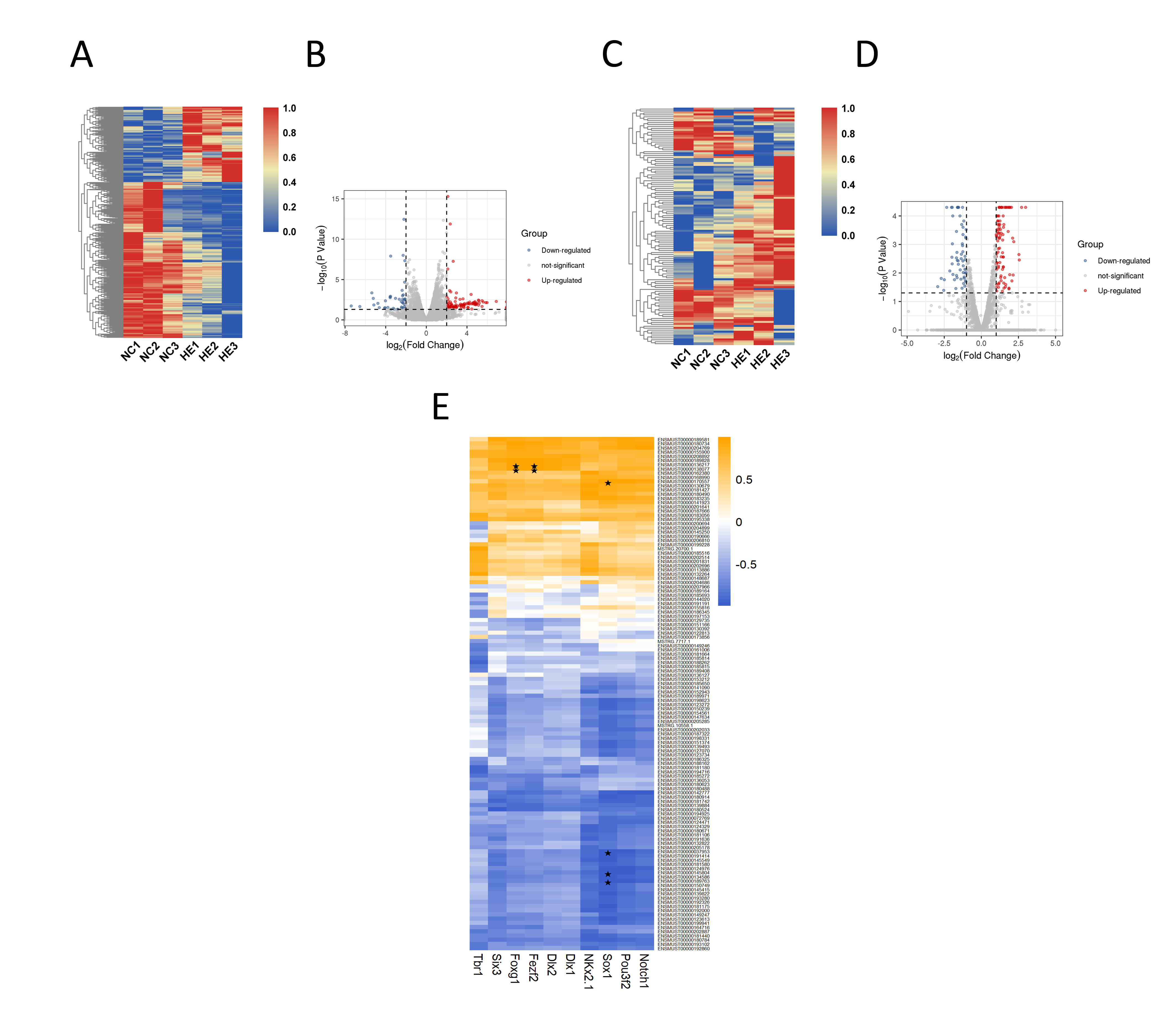

Supplement: Supplementary Figure S2 — DE gene identification and DE lncRNA-Hub mRNA interaction network analysis using log2 (fold change) ≥1 or ≤−1 with p < 0.05 as the cutoff. (A) Heatmap of 567 DE mRNAs in HE NSC/NPCs compared to NC. Red indicates upregulation, and blue indicates downregulation; row scale is from 0 to 1. (B) Volcano plot of DE mRNAs in HE NSC/NPCs compared to NC, red dots represent 383 upregulated mRNAs, and blue dots represent 184 downregulated mRNAs. (C) Heatmap of 148 DE lncRNA transcripts in HE NSC/NPCs compared to NC. Red indicates upregulation, and blue indicates downregulation; row scale is from 0 to 1. (D) Volcano plot of DE lncRNA transcripts in HE NSC/NPCs compared with NC. Red dots represent 89 upregulated transcripts, and blue dots represent 59 downregulated transcripts. (E) Heatmap of DE lncRNA-Hub mRNA PCC score. Orange indicates positive PCC, and blue indicates negative PCC. PCC ≥0.990 or ≤−0.990 and p < 0.05 are labeled with ⋆. [file Image_2.tif]

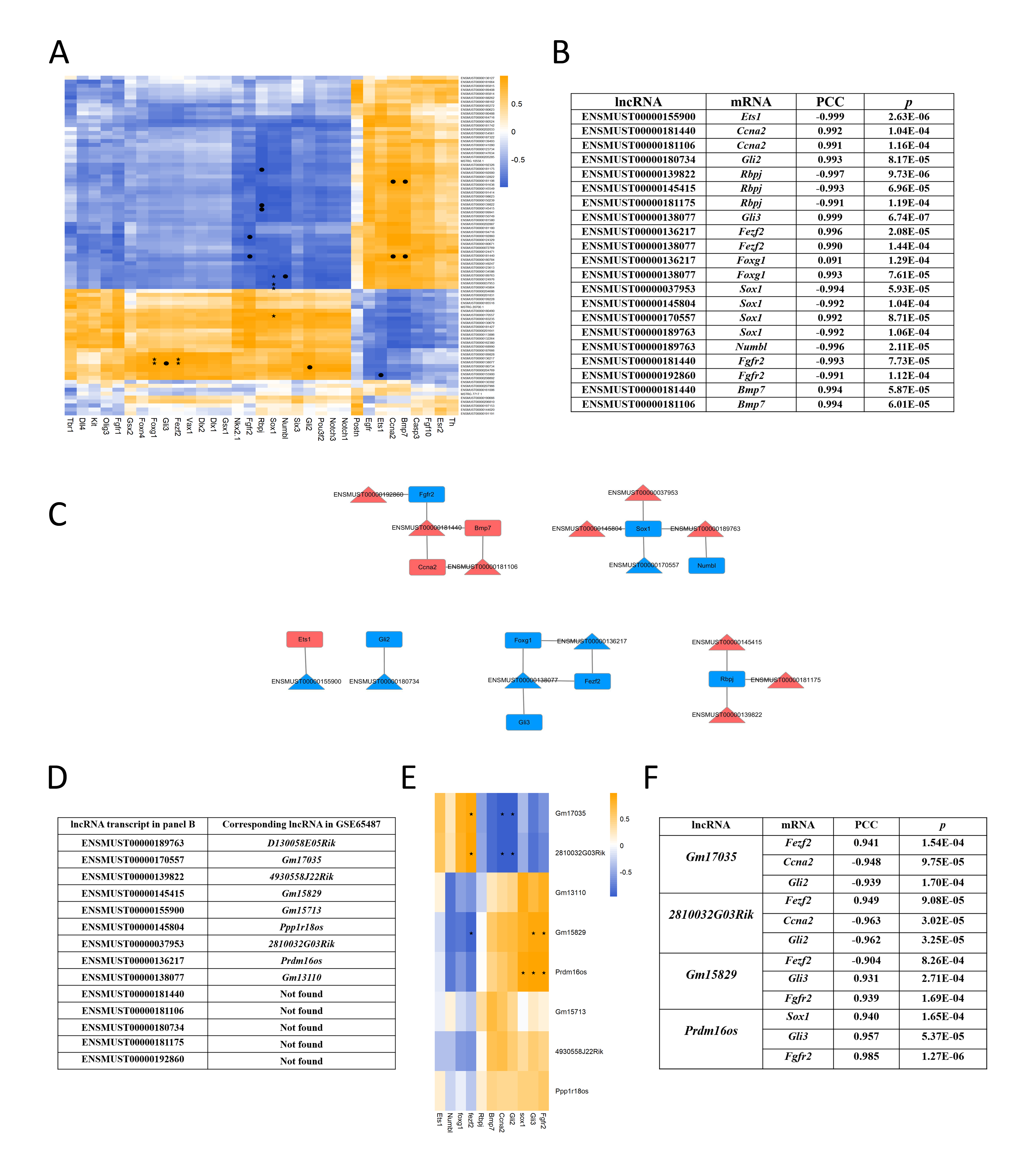

Supplement: Supplementary Figure S3 — DE lncRNA-core mRNA interaction network construction and verification with public dataset. (A) Heatmap of DE lncRNA-core mRNA PCC score. Orange indicates positive PCC, and blue indicates negative PCC. PCC ≥0.990 or ≤−0.990 and p < 0.05 are labeled with ⋆ (DE lncRNA-Hub mRNA) or ● (DE lncRNA-non Hub mRNA). (B) List of correlated DE lncRNA transcript-core mRNA pairs, with their PCC and value of p. (C) The interaction network of 14 DE lncRNA transcripts and 11 core mRNAs. Red indicates upregulation, and blue indicates downregulation in HE NSC/NPCs compared to NC. Triangles represent lncRNAs, and rectangles represent mRNAs. (D) List of lncRNAs in panel B with the corresponding lncRNA genes found in GSE65487. (E) Heatmap of lncRNA-mRNA PCC score graphed using data from GSE65487. Orange indicates positive PCC, and blue indicates negative PCC. PCC ≥0.900 or ≤−0.900 and p < 0.05 are labeled with ⋆. (F) List of correlated lncRNA-mRNA pairs presented in panel E, with their PCC and value of p. [file Image_3.tif]
